# Safety of Dietary Guanidinoacetic Acid: A Villain of a Good Guy?

**DOI:** 10.3390/nu14010075

**Published:** 2021-12-24

**Authors:** Sergej M. Ostojic

**Affiliations:** 1 Department of Nutrition and Public Health, University of Agder, 4604 Kristiansand, Norway; sergej.ostojic@uia.no; Tel.: +47-38-14-13-64; 2 FSPE Applied Bioenergetics Lab, University of Novi Sad, 21000 Novi Sad, Serbia

**Keywords:** toxicity, methylation, hyperhomocysteinemia, creatine, neuromodulation, MCDA

## Abstract

Guanidinoacetic acid (GAA) is a natural amino acid derivative that is well-recognized for its central role in the biosynthesis of creatine, an essential compound involved in cellular energy metabolism. GAA (also known as glycocyamine or betacyamine) has been investigated as an energy-boosting dietary supplement in humans for more than 70 years. GAA is suggested to effectively increase low levels of tissue creatine and improve clinical features of cardiometabolic and neurological diseases, with GAA often outcompeting traditional bioenergetics agents in maintaining ATP status during stress. This perhaps happens due to a favorable delivery of GAA through specific membrane transporters (such as SLC6A6 and SLC6A13), previously dismissed as un-targetable carriers by other therapeutics, including creatine. The promising effects of dietary GAA might be countered by side-effects and possible toxicity. Animal studies reported neurotoxic and pro-oxidant effects of GAA accumulation, with exogenous GAA also appearing to increase methylation demand and circulating homocysteine, implying a possible metabolic burden of GAA intervention. This mini-review summarizes GAA toxicity evidence in human nutrition and outlines functional GAA safety through benefit-risk assessment and multi-criteria decision analysis.

## 1. GAA Physiology, Biomolecular Interactions and Pathways

**Figure 1:**
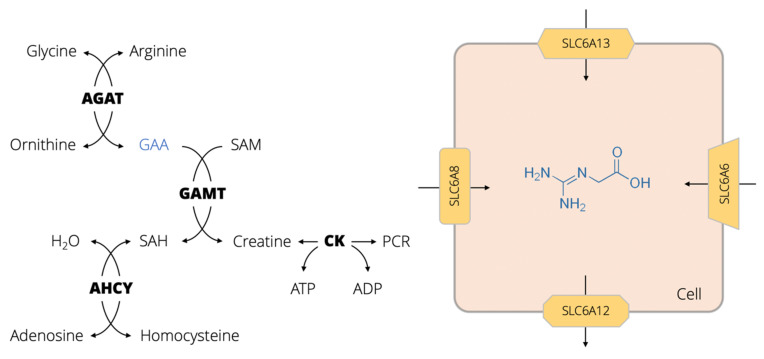
Metabolism and transport channels of guanidinoacetic acid (GAA). Abbreviations: AGAT, L-arginine:glycine amidinotransferase; SAM, *S*-adenosyl-L-methionine; GAMT, guanidinoacetate N-methyltransferase; SAH, *S*-adenosyl-L-homocysteine; CK, creatine kinase; PCR, phosphocreatine; AHCY, adenosylhomocysteinase; ATP, adenosine triphosphate; ADP, adenosine diphosphate. Yellow shapes depict different influx and efflux cellular transporters for GAA, including creatine transporter (SLC6A8), GABA transporter (SLC6A13), taurine transporter (SLC6A6), and 5-monocarboxylate transporter 12 (SLC16A12).

Guanidinoacetic acid (GAA, also known as glycocyamine, betacyamine or *N*-amidinoglycine) belongs to the class of organic compounds known as alpha amino acids and derivatives. GAA (chemical formula C_3_H_7_N_3_O_2_) is produced endogenously in the human body from non-essential amino acids glycine and arginine, in a reaction controlled by an enzyme *L*-arginine:glycine amidinotransferase (AGAT) (Figure 1). AGAT catalyzes the transfer of an amidino group (-C(=NH)NH_2_) from arginine to glycine to synthesize GAA, with ornithine as a byproduct. The reaction mainly takes place in the kidney, liver, and pancreas; however, GAA is also produced in the skeletal muscle, brain, and across the gut [1,2,3]. In the next step, GAA is combined with *S*-adenosyl-L-methionine, a reaction catalyzed by guanidinoacetate N-methyltransferase (GAMT), to produce creatine and *S*-adenosyl-L-homocysteine. The formation of creatine is a major metabolic fate of GAA, and AGAT-driven reaction is considered a rate-limiting step of creatine biosynthesis [4]. Since creatine is recognized as a critical molecular facilitator of cellular bioenergetics [5], the GAA synthesis-breakdown cycle thus remains of utmost importance for energy homeostasis. The role of GAA in the control and provision of cellular energy could also be highlighted by its interaction with cellular transporters for taurine (SLC6A6) and γ-aminobutyric acid (SLC6A13) [6,7], previously dismissed as un-targetable carriers by other bioenergetics therapeutics, including creatine (for a review, see Ref. [8]); monocarboxylate transporter 12 (SLC16A12) is involved in GAA efflux [9].

Plasma and urine GAA concentrations of 2.3 ± 0.8 μmol/L and 31.2 ± 21.7 mmol/mol of creatinine likely illustrate natural equilibrium in GAA metabolism [10]. However, several pathological conditions can affect GAA production-utilization circle, including kidney dysfunction, neurological diseases, or inherited metabolic disorders (for a detailed review, see Ref. [11]). Besides serving as an immediate precursor of creatine, GAA can also have several non-creatine-related metabolic roles, including the stimulation of hormonal release and neuromodulation, alteration of metabolic utilization of amino acids, vasodilation, and oxidant–antioxidant tuning (for a detailed review, see Ref. [12]). In addition, GAA could be obtained by a regular diet [13,14] and gut microbiota [15], yet these pathways contribute marginally to the total daily turnover of GAA.

## 2. GAA as a Dietary Agent in Human Nutrition

The first documented report of GAA utilization as an experimental nutritional intervention in humans arguaby dates back approximately 70 years ago. The group of Henry Borsook from Caltech University demonstrated the beneficial effects of GAA (combined with betaine) in treating cardiac decompensation [16]. The authors treated cardiac patients with a daily dosage of ~70 mg of GAA per kg body weight for up to 12 months, and many patients reported the so-called ‘sthenic effect’, comprising of an improved sense of wellbeing, less fatigue, and enhanced mental and physical performance. This was attributed to a GAA-driven recovery of phosphocreatine, the main reservoir of immediately available energy in energy-demanding tissues. The promising effects were soon corroborated in both hospital and ambulatory patients with heart disease, who reported feeling better after taking GAA, while the treatment produced no harmful effects, even when ingested over a long period of time [17]. A few patients with congestive heart failure were able to discontinue pharmacological treatment entirely while consuming GAA, without any unfavorable sequels [18]. Following these pioneering trials, GAA was intensively studied during the 1950’s in patients with arthritis and concurrent disease [19], acute and chronic poliomyelitis [20,21,22,23], myopathic muscular dystrophy [24], anxiety and depression [25], coronary arteriosclerosis [26], myasthenia gravis [27], motor-neuron disease [28], and neuromuscular disease [29]. Those historical studies were characterized by several methodological constraints and yielded equivocal results with regard to GAA efficacy, yet the supplementation with GAA was found harmless and non-toxic.

After this inception, subsequent decades prompted a relatively limited interest in studying dietary GAA until 1999, when a Japanese group put forward GAA as a nutritional supplement to compensate for a disease-driven GAA shortage in patients with chronic renal failure [30]. During the past decade, studies in healthy humans evaluated the effectiveness and safety of supplemental GAA when administered solely or combined with other nutrients [31,32,33], the dose-response effects of GAA [34,35], and the impact of dietary GAA on neuromodulation [36], exercise performance [37], oxidant–antioxidant capacity [38], skeletal muscle and brain bioenergetics [39,40], and epigenetic pathways [41]. Dietary GAA was also administered in women with chronic fatigue syndrome [42], or older adults [43], and put forward as a possible treatment in AGAT deficiency [44], and skeletal muscle medicine [45]. The contemporary studies mainly paralleled findings of the seminal trials from the early 1950’s, implying the advantageous effects of supplemental GAA on clinician- and patient-reported outcomes, now complemented by more robust study designs and an extensive list of pertinent biomarkers. In addition, recent trials suggested that GAA might be superior to creatine for improving bioenergetics in energy-demanding tissues [46], probably due to better transportability to target organs [8], and fewer non-responders compared to creatine [47]. This might be a rationale for its possible application in human nutrition, as an alternative or substitute of creatine, at least in some circumstances. Even so, GAA is still deemed as an experimental dietary additive since its utilization is not completely described in terms of efficacy, approval, labeling, and pharmacovigilance. At this moment, GAA can be found in several commercial formulations available in the U.S. and European markets, although no recorded standards of identity, quality, and corresponding analytical methods for GAA are currently available in the U.S., European, or Japanese pharmacopeias. The end-consumers might be, therefore, exposed to supplemental GAA while being unaware of possible safety issues.

## 3. Dietary GAA Safety and Toxicity

### 3.1. Methyl Group Depletion

Even the seminal paper that described the biochemical basis of GAA treatment recognized the possible risk of methyl group depletion following GAA consumption [16]. Since the transformation of GAA to creatine requires a donation of a methyl group (-CH3) from *S*-adenosyl-L-methionine, an excessive GAA intake can hypothetically drain the stores of methyl donors in the human body (e.g., methionine, choline, folic acid, B vitamins). The metabolic burden of methyl donor deficiency can perturb many cellular functions, including DNA methylation, neurotransmission, antioxidant defense, and protein synthesis [48]. Several human studies so far evaluated the risk of methyl group depletion after GAA intake. Our group investigated whether three different dosages of GAA (up to 4.8 g per day) administered for six weeks in healthy volunteers affect various serum and urinary variables related to GAA metabolism, including B vitamins [34]. We found that serum concentrations of folic acid, vitamin B6, B12, and holo-transcobalamin (carrier protein which binds the active form of vitamin B12) were not affected by the placebo or GAA intervention, implying that GAA dosages administered in this trial are probably insufficient to significantly impact circulating biomarkers of methyl donor micronutrients. Another trial evaluated the effects of supplemental GAA on DNA methylation [41], a critical epigenetic process for genome regulation. In this open-label, repeated-measure interventional trial, the authors evaluated the impact of 12 weeks of GAA supplementation (3 g per day) on global DNA methylation in healthy men and women. Dietary provision of GAA had no effect on DNA methylation, with 5-methylcytosine (a methylated form of the DNA base cytosine) non-significantly increased at post-administration, while a non-significant DNA hypomethylation was found in 3 of 14 participants. However, it remains unknown whether methyl group depletion that might be caused by dietary GAA affects other biological methylation pathways, including amino acid and protein methylations or polysaccharide methylation.

### 3.2. Hyperhomocysteinemia

*S*-adenosyl-l-homocysteine is a byproduct of GAA utilization that is further converted to homocysteine in a simple one-step reaction catalyzed by adenosylhomocysteinase: *S*-adenosyl-l-homocysteine + H_2_O <=> l-homocysteine + adenosine. Since hyperhomocysteinemia has been recognized as an independent risk factor for various cardiometabolic diseases [49], a possible augmentation of circulating homocysteine after GAA intake could thus be considered as a possible adverse effect of the intervention. Indeed, healthy young men and women who received 2.4 g of GAA per day for six weeks experienced a significant rise in serum homocysteine [31]. Elevated homocysteine levels were found in 55.6% of participants supplemented with GAA in another interventional trial [32], with a distinct dose-response effect of dietary GAA demonstrated for elevated serum homocysteine concentrations [34]. In the longest human study so far, supplemental GAA (3 g per day) significantly elevated serum homocysteine at 10-week follow-up (73.5% corresponding to 5.0 µmol/L), with 4 out of 20 participants (20.0%) experiencing clinically relevant hyperhomocysteinemia (>15.0 µmol/L) at post-administration [50]. This trial also revealed no effects of GAA on traditional biomarkers of cardiometabolic risk and inflammation, including HDL cholesterol, triglycerides, high-sensitive C-reactive protein, insulin, and ferritin. An atherogenic profile remained essentially unaffected by the intervention, indicating no major cardiometabolic burden of medium-term GAA intervention in healthy humans. Interestingly, co-administration of GAA and homocysteine-lowering agents can suppress or prevent a rise in homocysteine. No cases of elevated serum homocysteine were found in healthy human subjects supplemented with GAA and betaine (also vitamin B12, vitamin B6, and folic acid) [32]. Similarly, adding creatine to GAA largely prevented a GAA-driven rise in homocysteine in metabolically healthy men and women [33,51]. Having this in mind, dietary GAA should be carefully scrutinized as an experimental dietary additive due to its proven capacity to drive increased homocysteine production, which encourages its future utilization in human nutrition along with homocysteine-reducing agents.

### 3.3. Neurotoxicity

Several in vitro and animal studies documented neurotoxic effects of exogenously administered GAA. The group of Angela Wyse reported that the intrastriatal injection of GAA induced inhibition of Na+/K+-ATPase activity [52], glutamate uptake [53], and antioxidant defense in the rat brain [54]. GAA can affect brain cell development in rat brain cell cultures by causing axonal hyper-sprouting and decrease in natural apoptosis, followed by an induction of non-apoptotic cell death [55], suggesting that GAA may have different toxicity in the developing brain than in adults. Neu et al. [56] suggested that activating gamma-aminobutyric acid (GABA) receptors A might represent a candidate mechanism explaining neurological dysfunction induced by GAA. The accumulation of GAA in the brain was also found in children with inborn errors of creatine metabolism [57], suggesting that extra GAA might contribute to neurological complications in humans, such as epilepsy and seizures [58]. However, a recent study found that dietary GAA (up to 60 mg per kg body weight) does not accumulate in the brain of healthy men [59], with GAA levels remained essentially unchanged at eight-week follow-up when averaged across twelve white and grey matter locations. This study suggests that the GAA-driven neurotoxicity might be referenced to the level of GAA exposure, with the threshold of toxicological concern (although currently unidentified) highly unlikely to be acquired after dietary intake. For instance, brain GAA levels are 100–300 times higher than normal in experimental models with intrastriatal administration of GAA and/or inborn errors of creatine metabolism [57], which is substantially below GAA concentrations after dietary supplementation. Interestingly, GAA loading appears to affect peripheral GABA metabolism in healthy men and potentially down-regulates GABA synthesis in peripheral tissues [36]. Although safety sequels of this study are not elaborated further, GABA modulation should be considered as a possible neuromodulatory effect of dietary GAA.

### 3.4. Other Adverse Effects

An exposure to GAA in animal studies exacerbated ethanol-induced liver injury [60,61], stimulated osteoclastogenesis [62], generated reactive oxygen species [63], and modulated cerebral cortex potentials [64]. However, dietary GAA produced no effects on hepatic panel and cumulative action of antioxidants present in plasma after medium-term intake in healthy men and women [31,38]. Supplemental GAA can provoke a minor transitory gastrointestinal distress (e.g., intestinal cramping, bloating, abdominal pain) in healthy adults; the proportion of participants who reported gut-related side effects was not different from placebo [31]. GAA also induced a rise in serum creatinine due to an increase in creatine and subsequent degradation to creatinine. Creatinine is a surrogate marker of kidney damage, yet the other biomarkers of kidney damage remained unaffected by GAA intake [31].

## 4. Blueprint for GAA Risk-Benefit Assessment

**Figure 2:**
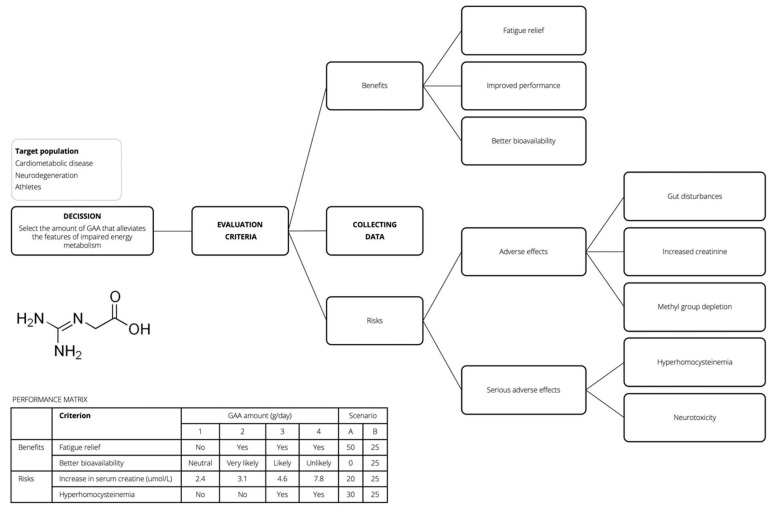
Risk-benefit tree and performance matrix for a hypothetical guanidinoacetic acid (GAA) case study.

The risk-benefit assessment is an emerging concept in the area of food safety [65,66], with the multi-criteria decision analysis (MCDA) advances as a new tool to estimate the hazards and advantages associated with the use of food interventions and dietary choices. MCDA combines heterogeneous research data into a compendious numeric that can be used to guide the selection of various health interventions in the context of risk-benefit profile [67]. In the case of GAA, the risk-benefit analysis could help in the pragmatic weighing of probabilities of side effect(s) against the benefit(s) as a consequence of GAA dietary exposure. Since GAA is an experimental dietary supplement, with only a handful of human studies available at this moment, MCDA could be created as a preliminary framework at best. In the first step (defining the decision problem), health professionals should establish the issue of interest, such as selecting the appropriate amount of supplemental GAA that safely alleviates the features of impaired energy metabolism. This step also involves nominating the target group, perhaps the clinical and general population, who can profit from energy-uplifting agents. The second step includes identifying the evaluation criteria (e.g., the prevalence of harmful health effects, improved brain function, reduced fatigue) against which various GAA dosages will be appraised. This step requires building a risk-benefit tree, based on feedback from experts and/or published trials (Figure 2). The third step of MCDA entails collecting data (e.g., qualitative, semi-quantitative, quantitative) on criteria selected above, and building the performance matrix describing how varying amounts of supplemental GAA perform when evaluated against each criterion. Only the effects with the highest level of evidence should be included in the final assessment, perhaps extracted from randomized controlled trials in the case of GAA. The fourth step defines the weights of the criteria, which enables setting up relative importance for each criterion depending on clinical significance using the ‘swing weighing’ approach [68]. For example, criteria weight for GAA-driven rise in serum homocysteine might include either quantitative scale (e.g., an absolute increase in µmol per liter) or qualitative method (e.g., using ‘yes’ or ‘no’ for inducing hyperhomocysteinemia); alternative scenarios representing different criteria-weighing schemes are also possible. Step five involves analyzing and synthesizing all risks and benefits while producing a single metric that can be used in grading GAA overall performance for efficacy-safety decisions; this requires accounting for uncertainty using statistical modeling [69]. The final sixth step includes reporting narrative and graphical results of an aggregated measure, enabling a decision-maker to rank alternative GAA dosages in terms of safety-efficacy profile. 

## 5. Conclusions

GAA is an investigational dietary supplement. Preliminary human studies suggest that dietary GAA has a relatively acceptable safety profile, yet medium-term intake appears to provoke unfavorable biochemical abnormalities, such as the rise in serum homocysteine (which could be attanuated by GAA co-ingested with creatine). Other adverse events demonstrated in animal studies with non-enteral administration of GAA are not confirmed in human trials with supplemental GAA thus far. Still, the possible toxic effects of GAA in the nervous system reported in pre-clinical research (e.g., modulation of GABA-ergic neurotransmission, impairment of brain cell development, epileptogenic activity) remain of high concern, with additional human studies required before advancing GAA for human use. Whether beneficial effects outcompete side effects of GAA currently remains unknown in terms of evidence-based efficacy and safety data. A comparative analysis of supplemental GAA safety using multi-criteria decision analysis remains highly warranted to optimize treatment selection within the settings of nutrition and clinical bioenergetics.
